# Genetic and epigenetic differentiation in response to genomic selection for avian lay date

**DOI:** 10.1111/eva.13703

**Published:** 2024-06-28

**Authors:** Melanie Lindner, Irene Verhagen, A. Christa Mateman, Kees van Oers, Veronika N. Laine, Marcel E. Visser

**Affiliations:** ^1^ Department of Animal Ecology Netherlands Institute of Ecology (NIOO‐KNAW) Wageningen The Netherlands; ^2^ Chronobiology Unit, Groningen Institute for Evolutionary Life Sciences (GELIFES) University of Groningen Groningen The Netherlands; ^3^ Wageningen University & Research (WUR) Wageningen The Netherlands; ^4^ Behavioural Ecology Group Wageningen University & Research (WUR) Wageningen The Netherlands; ^5^ Finnish Museum of Natural History University of Helsinki Helsinki Finland

**Keywords:** avian breeding time, climate change adaptation, DNA methylation, genomic selection, *Parus major*, SNPs

## Abstract

Anthropogenic climate change has led to globally increasing temperatures at an unprecedented pace and, to persist, wild species have to adapt to their changing world. We, however, often fail to derive reliable predictions of species' adaptive potential. Genomic selection represents a powerful tool to investigate the adaptive potential of a species, but constitutes a ‘blind process’ with regard to the underlying genomic architecture of the relevant phenotypes. Here, we used great tit (*Parus major*) females from a genomic selection experiment for avian lay date to zoom into this blind process. We aimed to identify the genetic variants that responded to genomic selection and epigenetic variants that accompanied this response and, this way, might reflect heritable genetic variation at the epigenetic level. We applied whole genome bisulfite sequencing to blood samples of individual great tit females from the third generation of bidirectional genomic selection lines for early and late lay date. Genomic selection resulted in differences at both the genetic and epigenetic level. Genetic variants that showed signatures of selection were located within genes mostly linked to brain development and functioning, including *LOC107203824* (*SOX3*‐like). SOX3 is a transcription factor that is required for normal hypothalamo‐pituitary axis development and functioning, an essential part of the reproductive axis. As for epigenetic differentiation, the early selection line showed hypomethylation relative to the late selection line. Sites with differential DNA methylation were located in genes important for various biological processes, including gonadal functioning (e.g., MSTN and PIK3CB). Overall, genomic selection for avian lay date provided insights into where within the genome the heritable genetic variation for lay date, on which selection can operate, resides and indicates that some of this variation might be reflected by epigenetic variants.

## 1 INTRODUCTION

Climate change has led to environmental changes at an unprecedented pace and, to persist, wild species need to adapt to their changing world. Data from long‐term study populations of wild animals have enabled the quantification of selection pressures and additive genetic variation for various phenotypes in many species and, based on these estimates, predictions of the potential for micro‐evolutionary responses to selection were derived (Grant & Grant, 1995; Kruuk et al., 2008). However, despite many examples of directional selection in the wild (Kruuk et al., 2002; Marrot et al., 2018; Nussey et al., 2005; Ramakers et al., 2019; Sheldon et al., 2003), micro‐evolutionary responses to selection that match the estimated predictions are rare (Charmantier & Gienapp, 2014; Merilä et al., 2001). Predicting micro‐evolutionary responses relies on accurate estimates of selection and genetic variation, but the complex nature of wild study systems and focal traits (and hence statistical frameworks) in combination with the limited data availability almost unavoidably leads to biased estimates of such parameters (but see the review by Pujol et al., 2018).

Advances in genomics provide us with the opportunity to zoom into the genomic architecture of phenotypes with the aim to identify the causal genes and genomic mechanisms that translate the relevant genes into phenotypes. This way, we can predict the potential for a micro‐evolutionary change to take place at specific genetic variants and derive predictions that take the genomic architecture of phenotypes into account. Indeed, loci that explained a moderate proportion of the genetic variation of phenotypes have been identified for some phenotypes, such as birth weight and recombination rate in ungulates (Johnston et al., 2016; Slate et al., 2002), wing length or bill morphology in birds (Bosse et al., 2017; Tarka et al., 2010) or age at maturity in salmon (Barson et al., 2015). Many phenotypes, however, are thought to be determined by a large number of genetic variants which each have a small effect on the phenotype (e.g., Husby et al., 2015; Santure et al., 2015). In combination with rather small sample sizes in most evolutionary studies of wild populations, small effects sizes lead to low power in detecting genetic variants, especially when phenotypes are phenotypically plastic, that is there is an environment dependent association between genetic variants and phenotypes (e.g., Gienapp et al., 2017). As a consequence, genetic variants only explain a small proportion of the heritable phenotypic variation for many phenotypes (e.g., references in table S1 of Gienapp et al., 2017), leaving a large proportion of the heritable phenotypic variation unexplained (or ‘missing’).

Epigenetics marks, that is chemical modifications of the DNA sequences or DNA‐associated histones, are suggested to contribute to heritable phenotypic variation (Trerotola et al., 2015). Epigenetic marks are involved in the regulation of gene expression and, as such, contribute to the expression of phenotypes. Especially if epigenetic marks are inherited independently of genetic variants or are involved in mediating genotype–environment interactions, they could, in theory, contribute to heritable phenotypic variation. While there are sources of epigenetics marks that are thought to be independent of genetic variation, such as the environmental induction of epigenetic modifications and spontaneous epi‐mutations (Sepers et al., 2019), it is under debate whether epigenetic variants that are independent of genetic variants can be inherited (especially in vertebrates). Nevertheless, the identification of epigenetic variants can provide insights into the genomic architecture of phenotypes, especially for phenotypes that are dependent on the environment. In maize, phenotypic plasticity in plant growth is suggested to be regulated by epigenetic processes that are driven by a genetic variant in the *rice plasticity 1* (*RPL1*) gene. Individuals that harbour the genetic variant in *RPL1* showed specific epigenetic signatures and increased levels of phenotypic plasticity (Zhang et al., 2012). In this case, epigenetic processes are responsive to the environment, but the degree of responsiveness is dependent on a genetic variant, indicating that the observed phenotypic plasticity is facilitated by genotype‐dependent epigenetic variation. Hence, specifically those epigenetic variants that are dependent on genetic variants are of particular interest for understanding how phenotypic variation is shaped.

Artificial selection is a promising and powerful tool for studying the genes and genomic mechanisms underlying natural variation of quantitative traits as it facilitates the comparison between individuals of extreme phenotypes at different molecular levels (Hill & Caballero, 1992). In contrast to phenotype‐based selection, genomic selection relies on the marker‐based prediction of genomic breeding values (GEBVs), the additive effect of an individual's genotype on the phenotype relative to the population mean phenotype (Charmantier et al., 2014). Despite its promising advantages and common application in animal and plant breeding (Jannink et al., 2010; Meuwissen et al., 2016), the only application of genomic selection in a wild animal population stems from the long‐term study population of great tits (*Parus major*) at the Hoge Veluwe National Park (Gienapp et al., 2019; Verhagen, Gienapp, et al., 2019).

In the Dutch study population of great tits, bidirectional genomic selection was applied to avian lay date, a trait with a heritability of about 0.2 (Gienapp et al., 2019). For this, GEBVs for lay date were estimated (Gienapp et al., 2019) and individuals with GEBVs for extremely early and late lay date were selected to establish the genomic selection lines (Verhagen, Gienapp, et al., 2019). The genomic selection experiment allows us to study the genetic and genomic basis underlying lay dates, a complex phenotype that is phenotypically plastic to spring temperature and of high evolutionary and ecological relevance for the population due to its effect on reproductive success (Schaper et al., 2012; Verhagen et al., 2020; Visser et al., 1998). The preparations for egg laying on a molecular and physiological level are initiated 6–8 weeks prior to the initiation of egg laying when increasing photoperiods in early spring induce a neuro‐endocrine cascade along the hypothalamic–pituitary–gonadal–liver axis (HPGL axis) that activates the onset of gonadal growth (Dawson et al., 2001; Williams, 2012). The final ‘decision’ on when to initiate egg laying is regulated much later in spring downstream of the underlying neuro‐endocrine cascade and depends on supplementary cues such as increasing temperatures (Caro et al., 2013; Schaper et al., 2012; Verhagen, Laine, et al., 2019). This complex regulation of lay dates across all levels of the HPGL axis in interaction with various environmental variables and the highly polygenetic nature of the trait have proven challenging for genome‐wide association studies (GWAS) on avian lay dates in wild populations. Even when moderate sample sizes are used (>2000 great tit females) and genotype–environment interactions are accounted for, GWAS failed to detect genome‐wide significant genetic variants for lay dates (Gienapp et al., 2017). As a consequence, the genetic and genomic basis underlying lay dates remain mostly unknown.

Genomic selection for lay dates has led to a clear differentiation in GEBVs accompanied by phenotypic differentiation when lay dates were recorded in aviaries (Verhagen, Gienapp, et al., 2019) and in the wild (Lindner et al., 2023). However, genomic selection based on GEBVs is a ‘blind process’ that does not provide any information on the genetic variants that responded to genomic selection or the epigenetic marks that accompanied the genetic response. Genetic signatures of selection within the genomic selection experiments were previously identified at a number of loci, in the form of single nucleotide polymorphisms (SNPs) (Verhagen, Gienapp, et al., 2019) and we here expand on these findings by exploring epigenetic signatures of the genomic selection line experiment. For this, we used a whole genome bisulfite sequencing (WGBS) approach on blood samples of female great tits from the third generation of the genomic selection lines for early and late lay dates to simultaneously profile heritable genetic variants and epigenetic marks in the form of SNPs and CpG site methylation (i.e., the methylation status of bi‐nucleotides within the DNA sequence that consist of a potentially methylated cytosine and an unmethylated guanine). We found differences at both the genetic and epigenetic level between females from the early and late selection line. Epigenetic variants were related to general biological processes, while genetic variants were related to brain development and functioning. This way, our findings provide insights into where within the genome heritable genetic variation for lay date resides and point towards complementary functions of genetic and epigenetic variants for avian lay dates.

## 2 METHODS

### 2.1 Genomic selection lines for avian lay date

The great tit, is a well‐known model species in ecology and evolution with a reference genome (Laine et al., 2016) and whole transcriptome and methylome for various tissues (Derks et al., 2016; Laine et al., 2016; Santure et al., 2011). The females included here originated from a bidirectional genomic selection experiment, in which great tits were selected based on GEBVs for early and late lay dates (full methods are provided in Gienapp et al., 2019; Verhagen, Gienapp, et al., 2019). We define a female's lay date as the date a female initiates egg laying within a year. In contrast to traditional methods for artificial selection where individuals are selected based on their expressed phenotype, genomic selection is based on genomic breeding values (GEBVs), the additive effect of an individual's genotype on the phenotype relative to the population mean phenotype (Charmantier et al., 2014).

For the genomic selection experiment, a wild training population of >2000 great tit females from the Hoge Veluwe National Park (The Netherlands) with known lay dates and genotyped at >500,000 single nucleotide polymorphisms (SNPs) was used to estimate genomic breeding values (GEBVs) using the ‘genomic best linear unbiased prediction’ (GBLUP) approach (Gienapp et al., 2019). Within this approach, the pedigree‐based relatedness matrix within the animal model was replaced by a SNP‐based relatedness matrix. The animal model constitutes a specific form of a mixed‐effect model frequently used in quantitative genetic studies (Wilson et al., 2010). Within this framework GEBVs of genotyped individuals with unknown lay date can be predicted based on the SNP‐based relatedness between individuals. To initiate the selection lines for early and late lay dates, 28 breeding pairs from the Hoge Veluwe study site were selected in 2014 as ‘parental’ generation based on their breeding values for lay dates (Verhagen, Gienapp, et al., 2019). All nestlings produced by the wild parental generation (i.e., the first‐generation offspring) were brought to aviary facilities at the NIOO‐KNAW on the tenth day after hatching (d10), where they were hand raised until independence (Drent et al., 2003). First‐generation offspring were genotyped (*n* = 163 first‐generation genotyped nestlings) to estimate their GEBVs and individuals with extreme early GEBVs and extreme late GEBVs were selected for breeding the selection lines for early and late lay dates in captivity (*n* = 37 first‐generation breeding pairs). Eggs produced by the first‐generation offspring in captivity were moved into nests of wild foster parents in spring 2015. Second‐generation offspring hatched in these foster nests and foster parents undertook the brood care until d10. Then, the nestlings were brought to the aviary facilities at the NIOO‐KNAW for hand rearing and genotyping (*n* = 189 second‐generation genotyped nestlings) and, based on the predicted GEBVs for lay date, selected into breeding pairs (*n* = 33 second‐generation breeding pairs) for spring 2016. This procedure was repeated for the third‐generation (*n* = 280 third‐generation genotyped nestlings). Overall, average GEBVs for third‐generation individuals (−0.50 and 0.61 for the early and late selection line, respectively) corresponded reasonably well to the cumulative predictive response to genomic selection of −0.72 days for the early selection line and 0.84 days for the late selection line (Verhagen, Gienapp, et al., 2019). The response to genomic selection at the level of GEBVs translated to a response at the phenotypic level when birds were breeding in aviaries (Verhagen, Gienapp, et al., 2019) as well as in the wild (Lindner et al., 2023). The experiment was performed under the approval by the Animal Experimentation Committee of the Royal Academy of Sciences (DEC‐KNAW), Amsterdam, The Netherlands, protocol NIOO 14.10.

### 2.2 Sample selection and DNA extraction

For the WGBS libraries, we selected 19 individual blood samples that included 10 samples from third‐generation females of the early selection line and nine samples from third‐generation females of the late selection line as well as one toe sample of a third‐generation female of the late selection line (Table S1). The toe sample was excluded from the DNA methylation analyses, but included for analyses on SNP data. We extracted DNA from blood samples (whole blood or blood of which plasma was removed) that were taken closest to the first of June in the first year of breeding. DNA was extracted from the samples using FavorPrep DNA extraction kit (Bio‐Connect, The Netherlands) following the manufacturer's instructions.

### 2.3 Whole genome bisulfite sequencing

Library preparation and sequencing was performed at the Roy J. Carver Biotechnology Centre (University of Illinois at Urbana‐Champaign, USA). WGBS data showed high rates of technical duplicates (Table S2) which can bias the methylation status and coverage of CpG sites. In contrast to RRBS, where the non‐random fragmentation complicates the differentiation between technical (e.g., PCR) and biological duplicates, the random fragmentation in WGBS allows for the removal of technical duplicates (for more details on technical duplicates see Laine et al., 2023). However, when duplication rates are high, the removal of technical duplicates results in a drastic loss of data. To circumvent data loss, we performed library preparation and sequencing twice for all libraries and a third time for eight libraries with particularly high duplication rates (Table S2). For the first two sequencing runs DNA from the same extraction was used and library preparation and sequencing were performed in the same way. Shotgun genomic libraries (with read length of 150 nucleotides) were prepared with the Hyper Library construction kit from kapa Biosystems (Roche) and treated with the EZ DNA Methylation‐Lightning kit from Zymo Research. Libraries were quantitated by qPCR and sequenced for 151 cycles from each end of the fragment (i.e., paired‐end) in two lanes of a S4 flow cell on a NovaSeq 6000. See Table S3 for how libraries were divided across lanes. For the third run, we extracted more DNA from eight of the previously sequenced samples using the same extraction protocol. Libraries were prepared as described above, pooled into one pool and sequenced for 151 cycles from each end of the fragment (i.e., paired‐end) in one lane of a SP flow cell on a NovaSeq 6000.

### 2.4 Bioinformatics processing

We ran the bioinformatics pipelines with snakemake v5.17.0 (Koster & Rahmann, 2012). We used R v4.0.1 (R Core Team, 2021) for additional scripts used within the pipeline and, in addition to base R packages, we used dplyr v1.0.0 (Wickham et al., 2020), tidyr v1.1.0 (Wickham & Henry, 2020), stringr v1.4.0 (Wickham, 2019), ggplot2 v3.3.2 (Wickham, 2016, p. 2), cowplot v1.1.0 (Wilke, 2020) and RColorBrewer v1.1.2 (Neuwirth, 2014) for data formatting and visualization. Environments were build and managed with conda v4.8.4 (Anaconda Software Distribution, 2016).

### 2.5 Quality control of whole genome bisulfite sequencing data

For the initial quality control, we used FastQC v0.11.9 (Andrew, 2010), FastQ Screen v0.11.1 (Wingett & Andrews, 2018), and MultiQC v1.7 (Ewels et al., 2016) in default settings but allowed parallel processing of samples by FastQC. Results are presented in Table S2. We trimmed the data and removed adapters using TrimGalore v0.6.5 (https://github.com/FelixKrueger/TrimGalore) in settings for paired‐end data and set a NovaSeq specific quality cut‐off of 20 (by specifying –2coulor 20) accounting for NovaSeq specific over‐representation of Gs (poly‐G). We repeated the quality control by running FastQC and MultiQC for the trimmed data.

### 2.6 Methylation calling

We used Bismark v0.22.3 (Krueger & Andrews, 2011) for alignment and methylation calling. First, the great tit reference genome build 1.1 (https://www.ncbi.nlm.nih.gov/assembly/GCF_001522545.3) was in silico bisulfite converted and indexed with default settings using Bismark's genome build function. We aligned the reads with default settings for paired‐end reads and set the number of parallel instances to be run concurrently to eight. We used the percentage of CHH methylation from the Bismark alignment reports to calculate the minimal bisulfite conversion efficiency (Table S4). We deduplicated the alignments with default settings for paired‐end reads using Bismark. Using Picard v2.23.3 (https://github.com/broadinstitute/picard) we added read groups to the alignments and merged the alignment files for either the first two sequencing runs or all three sequencing runs of the same sample depending on whether a sample was run two or three times. We assessed the number of mapped reads, average coverage depth and breadth of coverage using samtools (Table S5). The breadth of coverage was calculated as the number of bases with a minimum coverage of 10 divided by the total number of bases within the great tit genome, that is genome length; calculated using Bowtie2 v2.3.5.1 (Langmead & Salzberg, 2012). We used Bismark to call methylation from the deduplicated and merged alignments using default setting for paired‐end reads ignoring the first two and three bases from the 5′ end (of both reads) and the 3′ end (of both reads), respectively.

In addition to methylation called from the merged alignments (which are analysed in this study), we also called methylation from the alignments prior to merging, that is from alignment files for either the first two sequencing runs or all three sequencing runs of the same sample, using the pipeline described above. We used the derived methylation calls to assess whether our findings are robust in regard to the use of multiple sequencing runs.

### 2.7 Processing and analysis of methylation data

We used R package MethylKit v1.16.1 (Akalin et al., 2012) to import the raw methylation counts (Bismark output; CpG reports) into R. We excluded CpG sites with a CpG site coverage higher than the 99.9 percentile from further analyses. We combined methylation data of all samples into one data frame using MethylKit while merging cytosines from both strands of a CpG site into one CpG site by calculating the sum of methylated and unmethylated cytosines. We used custom R code to format the data for downstream analysis in which we removed CpG sites that did not have a coverage of 10x in all samples and removed CpG sites with 0% or 100% methylation across all samples (resulting in a total of 1,334,373 CpG sites).

### 2.8 Differential methylation analysis on CpG sites

We used the R package MethylKit to perform a differential methylation analysis on each CpG site to test for differences in DNA methylation between third‐generation females of the early (*n* = 10) and late (*n* = 9) selection line. To calculate the differential methylation statistics, we use the calculateDiffMeth function which calculates differential methylation using a Fisher's Exact test. Within the function call we applied overdispersion correction (overdispersion set to ‘MN’) as proposed by (McCullagh & Nelder, 1989). We validated the model by visually inspecting the *p*‐value distribution, the relationship of *p*‐values as ‐log_10_(*p*‐value) with CpG site coverage, the correlation of observed *p*‐values with the expected *p*‐values (both as ‐log_10_(*p*‐value), QQ‐plot) and the relationship of *p*‐values as ‐log_10_(*p*‐value) with the differences in mean methylation level between the selection lines (volcano plot). To identify differentially methylated sites (DMS), we corrected for multiple testing by using two adjustment methods. We (i) applied Benjamini and Hochberg's method implemented within MethylKit that controls the false discovery rate (FDR), the expected proportion of false discoveries within the rejected hypotheses (Benjamini & Hochberg, 1995), using a *q*‐value threshold of 0.05. We (ii) applied the Bonferroni approach using a corrected α‐threshold that was calculated as the initial α‐threshold (of 0.05) divided by the number of tests performed (i.e., 1,334,373 CpG sites). Validation plots of the differential methylation analysis show that, overall, the model was well‐suited for the methylation data (Figure S1a–d), although the QQ‐plot indicates some *p*‐value inflation (λ = 1.15; Figure S1c). Moreover, for DMS that passed the Bonferroni α‐threshold we showed that the identification of those DMS is not a statistical artefact of combining multiple sequencing runs by repeating the differential methylation analysis for the first and second sequencing run separately which, in both cases, confirmed our findings (Table S6).

In addition to the CpG site‐based analysis, we tested for a difference in overall methylation level across CpG sites between females from the early and late selection line using the CpG sites that passed the *q*‐value threshold. For this, we used the following generalized linear mixed model with binomially distributed errors implemented with the R package lme4 v1.1.27 (Bates et al., 2015);

(1) $$\;y=\mu +{\mathrm{\beta x}}_{\text{Line}}+S_{i}+F_{j}+e_{\mathrm{ij}}$$

with y as dependent variable, a two‐column matrix of methylated and unmethylated counts to account for variation in CpG site coverage (corresponding to methylation levels weighted by the total number of counts; Lea et al., 2017; Zhang et al., 2016), with μ for the intercept term, with β for the selection line regression coefficient, with *S*_*i*_ and *F*_*j*_ for the random effect terms of CpG site and female identity respectively (for *i* = 1.37 and *j* = 1.19) and with *e*_*ij*_ for the residual term. Significance was assessed by comparing the full model to its corresponding null model, that is a model equivalent to the full model but without the selection line regression coefficient, using an ANOVA.

### 2.9 Differential methylation analysis on regions

To identify differentially methylated regions, we predicted CGIs using cpgplot (Larsen et al., 1992), a software part of the EMBOSS package v6.6.0.0, in default settings. We predicted a total number of 33,142 CGIs and matched the 1,334,373 CpG sites that passed data processing to GCIs using BEDtools v.2.26.0 (Quinlan & Hall, 2010). We applied a filter threshold of 10 or more CpG sites (with 10x coverage in all samples) per CGI, resulting in a total of 596 CGIs. We used MethylKit's regionCounts function to sum up methylated and unmethylated base counts for each CGI and tested for differences in DNA methylation between the early (*n* = 10) and late (*n* = 9) selection line using MethylKit's calculateDiffMeth function. We used the same settings in the function call and the same approach for multiple testing correction as described above for the CpG site‐based differential methylation analysis.

### 2.10 SNP calling

Based on our recent evaluation of SNP calling tools for bisulfite sequencing data (Lindner et al., 2022), we used the Bayesian wildcard strategy of CGmapTools v0.1.2 (Guo et al., 2018) for SNP calling. Due to the bisulfite treatment (conversion of C to T if C is unmethylated), the presence of Ts might indicate either Ts or Cs in the unconverted genome resulting in ambiguous genotypes. Wildcards are used to denote this ambiguity in predicted genotypes with Y referring to either T or C and R referring to either A or G. When both strands have high coverage, this ambiguity can be resolved and an exact genotype can be computed (Guo et al., 2018). Prior to SNP calling, however, we were required to repeat the alignments as Bismark implemented new flag values (from v0.8.3 onwards) while CGmapTools requires the previous flag values for SNP calling. For the Bismark alignments with ‘old’ flag values we aligned the reads with the settings described above and additionally specified ‘–old_flag’ and ‘–no_dovetail’. We deduplicated the alignments, added read groups and merged alignments as described above (see *Methylation calling*). As females are the heterogametic sex, there is only one copy of the Z chromosome while autosomes have two copies. Here, we split the merged alignments such that the Z chromosome and mitochondrial DNA were removed and only autosomes and scaffolds were kept. We converted the alignments into ATCGmaps while removing the overlap of read pairs using CGmapTools. We called SNPs from the ATCGmaps using CGmapTools' Bayesian wildcard strategy in default settings. We removed ambiguous genotypes and applied a filter for minimum and maximum coverage using GATK v4.2.0 (DePristo et al., 2011; McKenna et al., 2010). We set the threshold for minimum coverage to 10 and the maximum coverage threshold to the 99th percentile of coverage within a sample averaged across sequenced samples. We merged samples, selected only positions that were SNPs and created unique SNP identifiers using GATK and BCFtools v1.11 (Danecek et al., 2021). The merged SNP data sets included 10,546,938 SNP positions prior to quality control.

### 2.11 Processing and analysis of SNP data

We used Plink v1.9 (Purcell et al., 2007) to manage SNP data and GenABEL v1.8.0 (Aulchenko et al., 2007) to perform the quality control. We discarded SNP positions that were present in less than 90% of samples per selection line and had a minor allele frequency below 0.125 (corresponding to five alleles), reducing the number of SNP positions to 451,600. We calculate the identity‐by‐state‐based genetic distance matrix for all samples over all SNPs which we then used for multi‐dimensional scaling (MDS, also known as principal coordinates analysis; Gower, 1966) to identify outliers and clusters of genetically similar individuals. No clear outlier was detected and samples clustered by selection line alongside the second PC (explaining for 8.84% of SNP variation; Figure S2 and Table S7).

The calculation of GEBVs for the genomic selection line experiment (Gienapp et al., 2019) was based on SNPs from a high‐density SNP chip for the great tit that covers >500,000 SNPs (Kim et al., 2018). SNPs from the SNP chip of females from all generations in the genomic selection line experiment (parents: *n* = 40, first‐generation offspring: *n* = 158, second‐generation offspring: *n* = 184, third‐generation offspring: *n* = 277), that after quality filter included 437,271 SNPs, were previously used for fixation index (*F*_st_) outlier analysis (Verhagen, Gienapp, et al., 2019). There was an overlap of less that 15% (*n* = 31,423) between the SNPs covered on the SNP chip (*n* = 437,271) and SNPs called from WGBS data (*n* = 451,600) and hence we consider our *F*_st_ outlier analysis complementary to the *F*_st_ outlier analysis performed in Verhagen, Gienapp, et al. (2019).

### 2.12 SNP outlier detection

To identify potential SNPs that differentiated in response to genomic selection for early and late lay dates, we performed three analytical approaches for SNP outlier detection described below; (i) fixation index (F_st_) outlier analysis, (ii) a genome scan for selection based on principle component analysis (PCA) and (iii) redundancy analysis. For SNPs without missing data, we defined outlier SNPs as SNPs that were detected as outliers in all three approaches. The redundancy analysis required to exclude SNPs with missing genotypes. Hence, for SNPs with missing genotypes we defined outlier SNPs as SNPs that were detected as outliers in the *F*_st_ outlier analysis and the PCA‐based genome scan for selection.

### 2.13 Fixation index (*F*_st_) outlier analysis

We estimated SNP‐specific *F*_st_ coefficients for SNPs called from WGBS data (*n* = 451,600) using BayeScan v2.1 (Foll & Gaggiotti, 2008), a method that aims to directly estimate the locus‐specific probability that SNPs are subject to selection using a Bayesian method. While the use of BayeScan can result in a high number of false positives, this is not the case when a large number of neutral loci (relative to selected loci) is included in the analysis (Lotterhos & Whitlock, 2014), which is the case here. We first converted SNP data into a BayeScan‐readable format using R packages adegenet v2.1.5 (Jombart, 2008; Jombart & Ahmed, 2011) and dartR v1.9.9.1 (Gruber et al., 2018). We implemented BayeScan with a few adjustments relative to the default settings. We (i) increased the prior odds to 100 (default: 10) as low prior odds lead to false positives when testing a large number of markers, we (ii) increased the number of iterations to 25,000 (default 5000) and the number of the thinning interval size to 50 (default: 10) to decrease autocorrelation between iterations while keeping the same number of iterations as in default settings, and we (iii) set the number of threads used to 60. Trace plots indicate good convergence of chains (Figure S3) and effective sample sizes to estimate the posterior distributions were sufficiently large (*n*_eff_ > 7000 for loglikelihood and *n*_eff_ > 13,000 for *F*_st_ coefficients). We considered SNPs with *q*‐value < 0.05 as *F*_st_ outliers. In addition to BayeScan, we estimated *F*_st_ using Arlequin and plink. For *F*_st_ outlier analysis with Arlequin v3.5.2 (Excoffier & Lischer, 2010) we followed the analysis described in Verhagen, Gienapp, et al. (2019) and considered SNPs with a *p*‐value < 0.01 as potential *F*_st_ outlier. For estimating locus‐specific *F*_st_ coefficients with plink we set the –fst and –within flags in combination with a file linking sample IDs to the selection line. Overall *F*_st_ coefficients estimated with BayeScan were lower than estimated with Arlequin and plink (Figure S4). However, the order of SNPs in regard to *F*_st_ coefficients is quite comparable between tools, specifically for SNPs with the highest *F*_st_ coefficients that correspond to *F*_st_ outliers.

### 2.14 Genome scan for selection based on principle component analysis (PCA)

We used the R package pcadapt v4.3.3 (Luu et al., 2017) to implement a genome scan for selection based on PCA under the assumption that SNPs that excessively relate to population structure are candidate SNPs for local adaptation. pcadapt performs two successive tasks, (i) a PCA on the centred and scaled SNP matrix and (ii) the computation of test statistics and *p*‐values based on the correlations between SNPs and the first K principal components (PCs). For our data set (*n* = 451,600 SNPs), we set K = 1, because only the first PC was significantly associated with selection line (*p*‐value = 2.04E‐10, all other PCs had *p*‐value > 0.05) otherwise and used default settings. SNPs with *p*‐values smaller that the Bonferroni‐based threshold (calculated as the initial α‐threshold of 0.05 divided by the number of tests performed, that is 451,600 SNPs) are considered PCA‐based outliers.

### 2.15 Redundancy analysis

We implemented a redundancy analysis (Forester et al., 2016) that aims to detect SNP outlier that constitute footprints of divergent selection typically expected in polygenetic adaptation when traits are in complex interactions with the environment (Caizergues et al., 2022). Redundancy analysis requires complete data and hence we removed SNPs with missing genotypes from the data set, reducing the data set to 164,959 SNPs with genotypes in all samples. We implemented the redundancy analysis with R package vegan v2.5.7 (Oksanen et al., 2019) and specified selection line as explanatory variable. We considered a relationship between SNP data and selection line with *p*‐value < 0.05 as significant and extracted the SNP‐specific loadings in the ordination space to identify SNPs involved in local adaptation. SNPs that are located in the tails of the distribution of the SNP‐specific loadings (using a cut‐off of three standard deviations; Forester et al., 2016) are considered redundancy analysis outliers.

### 2.16 Annotation of CpG sites and SNPs

We annotated genomic regions using R packages GenomicFeatures v1.42.3 (Lawrence et al., 2013) and rtracklayer v1.50.0 (Lawrence et al., 2009). The genomic regions included the TSS region (300 bp upstream to 50 bp downstream of the annotated transcription start site), promoter region (2000 bp upstream to 200 bp downstream of the annotated transcription start site), gene body (exons and introns) and 10 kb up‐ and downstream regions (respective 10 kb regions adjacent to the gene body). CpG sites and SNPs were matched to the above‐described annotated genomic regions of genes with BEDtools or assigned to the intergenic region (if no match was found).

After pre‐processing the methylation data sets, we retained 1,334,373 CpG sites that were associated to 17,917 annotated genes. Mostly CpG sites were located within the gene body (*n* = 773,206) of annotated genes or in intergenic regions (*n* = 421,872; Figure S5 and Table S8). Moreover, 209,466 CpG sites were located in the 10 k upstream region, 186,300 CpG sites in the 10 k downstream region, 58,770 CpG sites in the promoter region and 8738 CpG sites in the transcription start site region.

### 2.17 Gene enrichment analysis

We performed gene ontology (GO) analyses for gene lists identified with the statistical analysis of the methylation data using the ClueGO v2.5.7 (Bindea et al., 2009) plug‐in for Cytoscape v3.8.2 (Shannon, 2003). We used the human (v20.01.2022) and chicken (v26.01.2022) annotations, GO categories ‘biological process’, ‘cellular components’, ‘molecular function’, ‘immune system process’ and KEGG pathways, and custom background lists of all annotated genes within the methylation data (*n* = 17,916 genes). We specified the selection criteria for GO terms such that >=5% of the genes associated with a GO term and >=2 genes associated with the GO term had to be present in the input genes. We used a two‐sided enrichment/depletion test, *p*‐value correction for multiple testing via Bonferroni step down and set the network specificity to ‘medium’ ranging from the third to tenth GO level. The great tit annotation contains LOC genes and we checked whether the LOC genes were categorized as predicted genes, uncharacterized genes or small nuclear RNA and assessed the reliability of the gene prediction using the NCBI genome browser and blast (Table S9).

## 3 RESULTS

### 3.1 Differential methylation analysis

**FIGURE 1:**
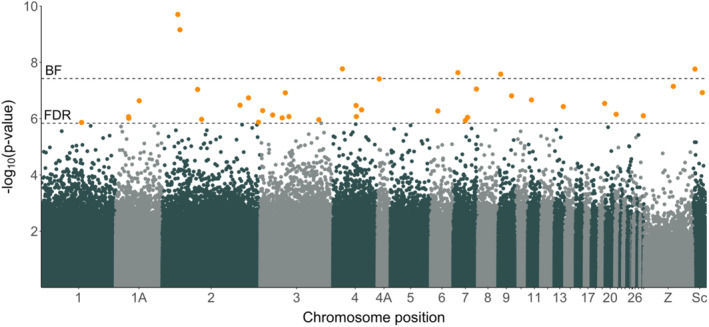
Manhattan plot of the differential methylation analysis. Data points are *p*‐values (on the ‐log_10_ scale) of individuals CpG sites (*n* = 1,334,373) that correspond to the significance of differential CpG site methylation between females from the early (*n* = 10) and late (*n* = 9) selection line. Black dashed lines refer to the significance threshold with Bonferroni (top) and FDR (bottom) correction for multiple testing. Furthermore, CpG sites that pass the FDR‐based significance thresholds are highlighted in orange and plotted with increased plot symbol size. ‘Sc’ refers to unplaced scaffolds (x‐axis).

**FIGURE 2:**
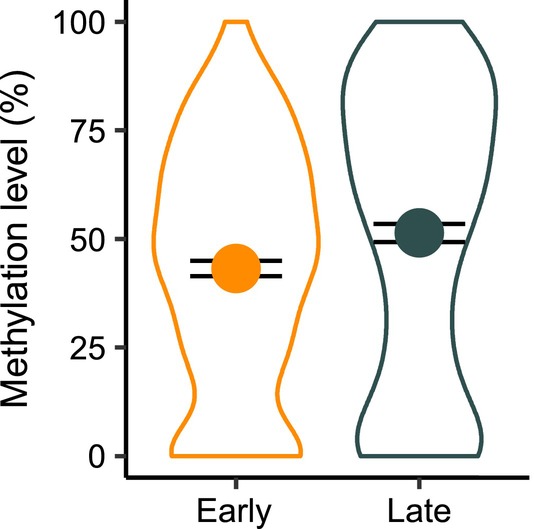
CpG site methylation level (in %) of 37 CpG sites with differential methylation after FDR correction for multiple testing for females from the early and late selection line. Data point and error bars represent the selection line‐specific mean and 95%‐confidence interval. The difference between selection lines was assessed using a likelihood ratio test (Chisq = 37.44, Df = 1, *p*‐value = 9.43*10^−10^).

Of the 1,334,373 CpG sites included in the CpG site‐based differential methylation analysis, we identified 37 differentially methylated CpG sites (DMS) when using the FDR‐based significance threshold (Figure 1). However, three DMS showed less than 10% difference in CpG methylation between selection lines (Table S10). Overall, identified DMS were hypomethylated in females of the early selection line relative to the late selection line (LRT: Chisq = 37.44, Df = 1, *p*‐value < 0.001; Figure 2). When applying a more stringent significance threshold (i.e., Bonferroni) and, this way, decreasing the number of expected false positives, we identified six DMS in four genes with at least 19% difference in CpG methylation between selection lines (Figure 2 and Table 1). These DMS were located within the gene body (*n* = 3), the 10 kb upstream region (*n* = 1) and the promoter region (*n* = 1) of annotated genes (Table 1), whose potential functional relevance for avian lay dates is described in the discussion.

**TABLE 1:** CpG sites with differential methylation that pass the Bonferroni significance threshold.

Site	*p*‐Value	Methylation difference (%, late‐early)	Genomic region	Gene symbol
chr2_23971207	1.99E‐10	23.43	Gene body	*CASD1*
chr2_27152785	6.96E‐10	30.23	Intergenic	NA
chr4_14314088	1.70E‐08	−28.00	Intergenic	NA
chr7_7319671^a^	2.32E‐08	−22.00	10 kb upstream	*MSTN*
chr7_7319671^a^	2.32E‐08	−22.00	Gene body	*C7H2orf88*
chr9_3916309^a^	2.63E‐08	19.34	Promoter	*PIK3CB*
chr9_3916309^a^	2.63E‐08	19.34	Gene body	PIK3CB
Scaffold101_1793295	1.74E‐08	19.75	Intergenic	NA

*Note*: The CpG site identifier (chromosome and position on the chromosome), *p*‐value, difference in CpG site methylation, genomic region and gene symbol of the annotated gene are shown. A positive difference in CpG site methylation corresponds to hypomethylation in females from the early selection line relative to females from the late selections line and vice versa. Full name of genes: CAS1 domain containing 1 (*CASD1*); myostatin (*MSTN*); chromosome 7 C2orf88 homologue (*C7H2orf88*); phosphatidylinositol‐4,5‐bisphosphate 3‐kinase catalytic subunit beta (*PIK3CB*).

^a^ Marks CpG sites that are annotated to more than one genomic region.

Enrichment analysis of genes that overlap DMS (*n* = 25) provided limited insights, likely due to the low number of genes. Only if we decreased the number of genes required per GO term to be included in the enrichment analysis to two genes, we found enriched GO terms and hence findings should be interpreted with care. We found nine enriched GO terms using human GOs (Table S11A) and seven enriched GO terms using chicken GOs (Table S11B) with three GO terms shared between species‐specific GO terms. GO terms were linked to general biological processes, including protein kinase B (PKB) signalling, that is linked to two genes encoding for the phosphatidylinositol‐4,5‐bisphosphate 3‐kinase catalytic subunit beta (*PIK3CB*, an intracellular signal transducer enzyme) and myostatin (*MSTN*, a growth differentiation factor). DMS in the promoter region of *PIK3CB* and in the 10 kb upstream region of *MSTN* also pass the more stringent significance threshold.

We performed a region‐based differential methylation analysis on 596 CGIs and did not identify any differentially methylated CGIs.

### 3.2 SNP outlier analyses

We implemented three analytical approaches to identify potential candidate SNPs underlying genomic selection for early and late lay dates and focused on the overlap between approaches; (i) F_st_ outlier analysis, (ii) a genome scan for selection based on PCA, and (iii) redundancy analysis (Table S12).

**FIGURE 3:**
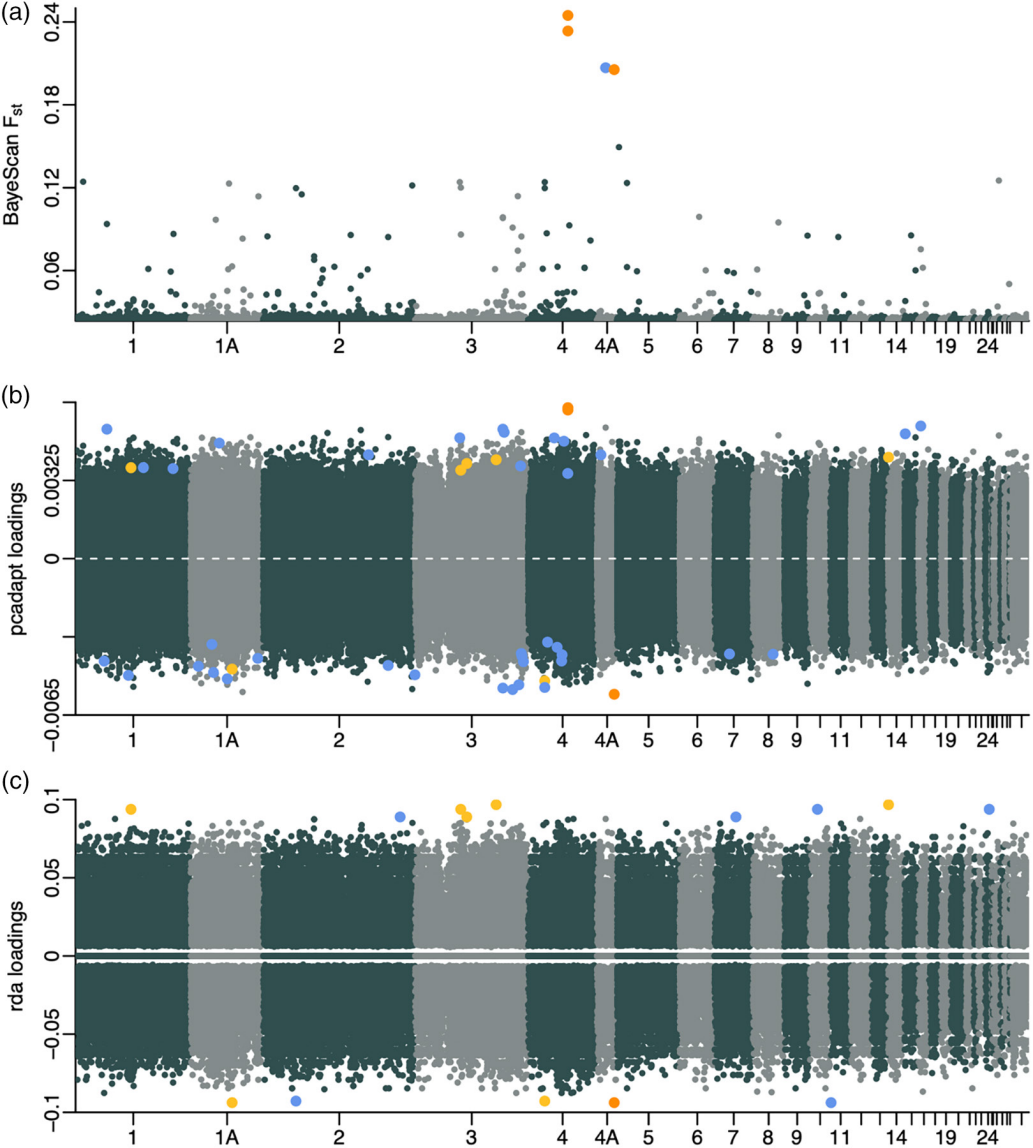
Manhattan plots for the SNP outlier analyses. *F*_st_ coefficients estimated with BayeScan (a), PCA‐based genome scan SNP loadings (b) and redundancy analysis SNP loadings (c) between females from the early (*n* = 10) and late (*n* = 10) selection line for each SNP (*n* = 451,600 for a and b, *n* = 164,959 for c). All SNPs above the significance thresholds are highlighted by increased plot symbol size. Outlier SNPs detected as outliers with all three approaches (or with BayeScan and pcadapt for SNPs with missing genotypes) are highlighted in orange. Tool‐specific outlier SNPs are highlighted in blue. SNPs detected as outliers with pcadapt and redundancy analysis are highlighted in yellow. ‘Sc’ (x‐axis) refers to unplaced scaffolds.

We estimated the F_st_ coefficients for 451,600 SNPs with BayeScan and identified four F_st_ outliers located on chromosomes 4 and 4A (*q*‐value < 0.05; Figure 3 and Table 2). While the two SNPs on chromosome 4 were located within intergenic regions, the two SNPs on chromosome 4A were located within the 10 kb upstream region of the transcription factor SOX‐3‐like gene (*LOC107203824*; *SOX3*‐like) and the gene body of the nik related kinase gene (*NRK*). *F*_st_ outliers and DMS showed no overlap in physical location on the genome or associated genes.

**TABLE 2:** Ooutlier SNPs.

SNP	*F*_st_	*q*‐Value	Allele frequencies	Genomic region	Gene symbol
chr4_39145992_T:C	0.24	2.40E‐04	E = 0.00:1.00, L = 0.85:0.15	Intergenic	NA
chr4_39145990_T:C	0.23	6.20E‐04	E = 0.00:1.00, L = 0.83:0.17	Intergenic	NA
chr4A_16892597_C:T	0.21	5.52E‐03	E = 0.25:0.75, L = 1.00:0.00	10 kb upstream	*LOC107203824* (*SOX3*‐like)

*Note*: For SNPs without missing data we defined outlier SNPs as SNPs that were detected as outliers in all three approaches. For SNPs with missing genotypes we defined outlier SNPs as SNPs that were detected as outliers in the *F*_st_ outlier analysis and the PCA‐based genome scan. The SNP identifier (chromosome, position and reference:alternative allele), *F*_st_ coefficient, *F*_st_ *q*‐value, allele frequencies (reference:alternative) for early (E) and late (L) selection line females, genomic region and gene symbol of the annotated gene are shown. Full name of gene: transcription factor SOX‐3‐like (*LOC107203824*; *SOX3*‐like).

We found a larger number of *F*_st_ outliers with Arlequin (*n* = 2230 with *p*‐value < 0.01), which is in line with the large number of *F*_st_ outliers (*n* = 4786 with *p*‐value < 0.01) previously identified in Verhagen, Gienapp, et al. (2019). When focusing on SNPs that are shared in both data sets (*n* = 31,423), 156 and 391 Arlequin *F*_st_ outlier SNPs remained for SNPs used here and SNPs used in Verhagen, Gienapp, et al. (2019), of which 46 SNPs are shared. This indicates that the significance threshold used (*p*‐value < 0.01) is rather tolerant for the number of SNPs tested. However, *F*_st_ outlier analysis with Arlequin and BayeScan performed here identified the same set of SNPs as outlier SNPs with the largest effect sizes (Figure S4).

The PCA‐based genome scan for selection on 451,600 SNPs implemented with pcadapt revealed that only the first PC was significantly associated with selection line (*p*‐value = 2.04E‐10, all other PCs *p*‐value > 0.05). Based on correlations between SNPs and the first PC, we identified 47 SNPs with outlier loadings.

In contrast to the *F*_st_ outlier analysis and the PCA‐based genome scan for selection, redundancy analysis cannot handle missing data which reduced the SNP data set to 164,959 SNPs. Redundancy analysis showed that selection line was indeed a significant explanatory variable (*F*‐statistic = 1.56, Df = 1, *p*‐value = 0.001) that explained 8.0% of the total variance in the SNP data. Based on the SNP‐specific loadings on the first axis, we identified 14 SNPs with outlier loading score (significance threshold: mean ± 3sd with mean = 1.20*10^−7^ and sd = 0.029, Figure 3).

When focusing on the overlap between approaches, we identified three outlier SNPs (SNP without missing genotypes: chr4A_16892597_C:T; SNPs with missing genotypes: chr4_39145992_T:C and chr4_39145990_T:C, Table 2) While the two SNPs on chromosome 4 were intergenic, the SNP on chromosome 4A was located within the 10 kb upstream region of *LOC107203824* (*SOX3*‐like).

## 4 DISCUSSION

Identifying the genetic variants and genomic pathways underlying complex quantitative traits is a major challenge in modern evolutionary biology. As many highly polygenic traits are expressed in interaction with the environment (e.g., Gienapp et al., 2017), this has proven challenging in wild populations. A unique genomic selection experiment for early and late avian lay date (Gienapp et al., 2019; Verhagen, Gienapp, et al., 2019) enabled us to identify both genetic and epigenetic differentiation between females of the early and late lay selection line and, this way, gain insights into the genetic variants and genomic pathways that might be involved in adaptation of a wild bird population.

### 4.1 Biological relevance of identified DMS and outlier SNPs

When comparing females from the early and late genomic selection lines for lay date, we found evidence for both epigenetic and genetic differentiation in response to genomic selection. Overall, differentially methylated CpG sites (DMS) and outlier SNPs were located within genes that have known functions for reproduction. The biological relevance of genes with DMS mostly concerned ovarian functioning (e.g., *PIK3CB*, *C7H2orf88* and *MSTN*), while the biological relevance of genes with outlier SNPs mostly concerned brain development and functioning (*LOC107203824*; *SOX3*‐like).

Genes with differential DNA methylation in response to genomic selection for lay dates mostly function downstream in the neuro‐endocrine system along the HPGL axis (i.e., in the ovary), indicating that genetic variation in DNA methylation potentially acts to fine‐tune lay dates (Lindner, Laine, et al., 2021; Verhagen, Laine, et al., 2019). Genetic differentiation, in contrast, was located within genes that mostly function at the stage where the neuro‐endocrine cascade is activated (i.e., upstream in the neuro‐endocrine system along the HPGL axis).

Genes with DMS have predicted functions in a variety of biological processes including ovarian and reproductive functioning, especially when focusing on DMS that passed the Bonferroni‐corrected significance threshold (*CASD1*, *MSTN*, *C7H2orf88* and *PIK3CB*). GO analysis pointed towards two genes encoding for an intracellular signal transducer enzyme (*PIK3CB*) and a growth differentiation factor (MSTN), that are of relevance for the protein kinase B (PKB) signalling pathway, a pathway associated with the survival of follicle cells. For example, inactivation of the PKB signalling pathway in chicken granulosa cells of the three largest preovulatory follicles led to oligonucleosome formation, which characterizes ovarian follicle atresia (Johnson et al., 2001). Although follicle atresia is a common process in pre‐hierarchical follicles, follicles that have been selected into the preovulatory hierarchy are committed to ovulate and rarely undergo follicle atresia (Johnson & Woods, 2009), highlighting the essential role for PKB‐mediated inhibition of follicle atresia in preovulatory follicles for ovarian functioning.

PIK3CB (p100β), together with PIK3CA (p100α) and PIK3CD (p100δ), builds the catalytic p110 subunit that characterizes a class of phosphatidylinositol 3‐kinase (PI3K) proteins that are primarily responsible for the production of phosphatidylinositol in response to growth factors (Cantley, 2002; Zhang et al., 2020). In general, PI3K signalling pathways play crucial roles in cell growth, cell survival and cell movement (Cantley, 2002), but are known to have more specific roles in reproductive functioning such as ovarian follicle development (Li et al., 2021). For example, PI3K signalling pathways in neuronal leptin receptors, that harbour catalytic subunits PIK3CA (p100α) and PIK3CB (p100β), are crucial for pubertal maturation and reproductive functioning in mice (Garcia‐Galiano et al., 2017, 2019) and the PIK3 and cAMP/protein kinase A (PKA) signalling is involved in the crosstalk between the transforming growth factor (TGF) β1 and follicle stimulating hormone (FSH) that mediates steroidogenesis in ovarian granulosa cells in rats (Chen et al., 2007). Furthermore, *C7H2orf88* encodes for the chromosome 7 C2orf88 homologue that is also known as *smAKAP*, encoding for the small membrane A‐kinase anchor protein. smAKAP constitutes a small protein that is directly anchored to membranes by acyl motifs and almost exclusively interacts with the type I regulatory subunits of cAMP/protein kinase A (PKA) signalling (Burgers et al., 2016), a key signalling pathway during the ovulation process (Shimada & Yamashita, 2011).

*MSTN*, also known as growth and differentiation factor 8 (*GDF8*) encodes a transcriptional growth factor that actively represses skeletal muscle growth (Kollias & McDermott, 2008; McPherron et al., 1997) and as such has been of much interest in livestock research (Bellinge et al., 2005). Genetic manipulation of *MSTN* in livestock resulted in increased muscle mass (e.g., the double‐muscled phenotype in cattle; Grobet et al., 1997), but compromised fertility, calf viability and stress susceptibility (Han et al., 2021). In quail, a three base‐pair deletion in *MSTN* resulted in amino acid deletion in the MSTN peptide and led to increased body weight and muscle mass (Lee et al., 2020) as well as delayed lay dates, higher egg weight and lower number of eggs produced during the active laying period (Lee et al., 2021) in homozygous mutant quail relative to heterozygous mutant and wild‐type quail. These studies in quail exemplify the relevance of *MSTN* for reproductive traits in seasonally breeding birds, including lay dates in quail.

*CASD1* (encoding for CAS1 domain containing 1) is also known as sialate O‐acetyltransferase (*SOAT*) that catalyses the 9‐O‐acetylation of sialic acids that constitute sugars at the reducing end of glycoproteins and glycolipids (Arming et al., 2011). SOAT has known to have an important role in lipid trafficking and utilization during embryonic development. For example, zebrafish embryos injected with a SOAT inhibitor showed a lower rate of yolk consumption indicating that SOAT is catalytically active in the yolk cholesterol trafficking during embryogenesis (Chang et al., 2016). In the chicken embryo, SOAT is responsible for the rapid esterification of a large proportion of yolk cholesterol, a process essential for lipid uptake from the yolk by the yolk sac membrane during embryonic development (Shand et al., 1993; Wang et al., 2017). While the role of SOAT in lipid trafficking and utilization during embryonic development within the egg is well understood for oviparous species, including birds, it is unclear whether SOAT is of any relevance for lipid deposition during the production of eggs.

For outlier SNPs, we focus on outliers detected with all three approaches and found one gene with an outlier SNP; *LOC107203824* (*SOX3*‐like). The transcription factor SOX‐3 encoded by *LOC107203824* (*SOX3*‐like) is crucial for the development and functioning of the hypothalamo‐pituitary axis (Rizzoti et al., 2004; Szeliga et al., 2021) which is an essential component of the hypothalamic–pituitary–gonadal–liver axis (HPGL axis). The HPGL axis mediates the neuro‐endocrine cascade that, in seasonally breeding birds, activates the onset of gonadal growth in response to increasing photoperiods (Dawson et al., 2001; Williams, 2012). Consequently, reproductive functioning is critically dependent on normal development and functioning of the hypothalamo‐pituitary axis. For example, a genetic variant in *SOX3* in humans was linked to normosmic idiopathic hypogonadotropic hypogonadism, a form of isolated gonadotropin‐releasing hormone (GnRH) deficiency (Kim et al., 2019). Next to the brain, *SOX3* is also expressed in the ovary where it is required for gonadal functioning. For example, double *SOX3* deletion in female mice led to excess follicular atresia, ovulation of defective oocytes, and severely reduced fertility (Weiss et al., 2003). SOX‐3 was also identified as one of eight master transcription factors driving folliculogenesis in mice (Bian et al., 2021) and was found to inhibit apoptosis during follicle development in zebrafish leading to improved fecundity (Hong et al., 2019). In general, *SOX3* constitutes a gene of high relevance for reproductive functioning, that is crucial for normal development and functioning of the hypothalamo‐pituitary axis.

### 4.2 Hypomethylation in the early selection line relative to the late selection line

The majority of DMS showed hypomethylation in females from the early selection line relative to females from the late selection line. However, the functional relevance of a general hypomethylation in females from the early selection line is unclear as CpG sites are located within many genes and within different genomic locations of genes. The association of CpG site methylation and gene expression is likely to differ between genomic features (Laine et al., 2016), making it difficult to derive general conclusions on the functional relevance. Considering a previously reported association between changes in DNA methylation throughout the breeding season and the onset of lay dates in great tit (Lindner, Laine, et al., 2021), it is possible that the observed hypomethylation in the early selection line is related to differences in seasonal changes in DNA methylation between the early and late selection line. However, the CpG sites at which DNA methylation is known to change with seasonality account for a small proportion of CpG sites (e.g., 35 of 5097 CpG sites (Lindner, Laine, et al., 2021)) and hence is unlikely to account for the overall hypomethylation in the early selection line observed here. Due to the lack of replicated selection lines, it is not possible for us to exclude the possibility that the overall hypomethylation in the early selection line is a consequence of genetic drift.

### 4.3 Epigenetic differentiation is hypothesized to be a correlated response to genetic differentiation following genomic selection

We here hypothesize that the observed differentiation in DNA methylation was accompanying the genetic differentiation induced by the genomic selection experiment. In other words, we hypothesize that the variants in DNA methylation were *inherited* with genetic variants that arose following genomic selection rather than inherited independently of genetic variants. While we were not able to explicitly test for an association between DNA methylation and genetic variants, this hypothesis is in line with findings by van Oers et al. (2020) where differences in DNA methylation between selection lines for exploratory behaviour in great tits were explained by genetic differences rather than spontaneous epi‐mutations. Furthermore, many recent studies have shown that a large proportion of variants in DNA methylation is indeed dependent on genetic variants. For example, in *Arabidopsis thaliana* the variation in CHH methylation in transposable elements was strongly associated with cis‐ and trans‐acting genetic variants (Dubin et al., 2015), in inter‐crosses between wild derived red junglefowl and domestic chickens >45% of mapped trait loci were controlled by five trans‐acting loci mainly associated with an increase in hypothalamic DNA methylation in red junglefowl genotypes (Höglund et al., 2020) and in great tit nestlings DNA methylation in early life is largely determined by genetic effects (common origin) rather than environmental effects (common rearing environment) (Sepers et al., 2023). This dependency of DNA methylation on genetic variation is also supported by studies showing that more closely related individuals are more similar in their methylation patterns than unrelated individuals (Lea et al., 2017; van Oers et al., 2020; Viitaniemi et al., 2019). There are, however, other sources of DNA methylation that concern the environmental induction of DNA methylation and spontaneous epi‐mutations. While such epi‐mutations are indeed established in plants (Feil & Fraga, 2012), there is limited evidence for their stable inheritance in vertebrates, or more specifically, in avian species (e.g., Sepers et al., 2019). In vertebrates, it is unclear how such epi‐mutations would escape the extensive reprogramming of DNA methylation that, for example in mammals, takes place during the fertilization of the zygote and in the primordial germ cell (the progenitors of sperm cells and oocytes; Seisenberger et al., 2013). Nevertheless, there are some comparative studies that imply a role for epigenetic variation in evolution, assuming that epigenetic variants are inherited independently of genetic variants. For example, epigenetic variants were more common among several closely related species of Darwin's finches than genetic variants in the form of copy number variation and there was no apparent overlap between epigenetic variants and genetic variants suggesting that epigenetic variants are distinct and correlate with the evolutionary history of Darwin's finches (Skinner & Guerrero‐Bosagna, 2009). Although it cannot be excluded that epigenetic variants are dependent on genetic variants, as this was not formally assessed, it is likely that epigenetic changes contribute to the molecular basis of the evolution of Darwin's finches. Hence, the study shows that DNA methylation might be inherited and contributes to evolution in its own right, that is independently of genetic variants, indicating that our interpretation of the observed differentiation in DNA methylation as a response that accompanied the genetic differentiation might be too simplistic.

### 4.4 Caveats and future challenges

We designed our study with great care, but there are some limitations that we need to address here. Firstly, our experimental design did not include any replicate lines (Verhagen, Gienapp, et al., 2019), limiting our ability to differentiate selection as the underlying cause of the reported epigenetic and genetic differentiation from genetic drift. We, however observe increased differentiation over generations between selection lines in GEBVs (genetic level) and lay dates (phenotypic level) (Lindner et al., 2023; Verhagen, Gienapp, et al., 2019), indicating that the genomic selection lines offer a suitable contrast between early and late avian lay date for the study of genetic variants and genomic pathways that might be involved in mediating avian lay date.

We succeeded to identify DMS and outlier SNPs of genome‐wide significance, but our statistical power was limited with a sample size of 9–10 females per selection line. This means that we were only able to identify DMS and outlier SNPs with very large effect sizes, resulting in an incomplete list of candidates DMS and outlier SNPs.

We hypothesize that the observed epigenetic differentiation arose as a consequence of genetic differentiation in response to genomic selection for lay date. While, in theory, it is possible to test for an association between genetic variants and epigenetic variants and estimate the proportion of variation in DNA methylation that is explained by genetic variation (Dubin et al., 2015; Höglund et al., 2020), our limited sample size did not provide us with the statistical power needed for such an analysis. To increase sample size, we could have chosen an alternative and more cost‐efficient sequencing approach, such as reduced representation bisulfite sequencing (RRBS) (e.g., Gu et al., 2011; Meissner et al., 2008). However, we did not have an a priori expectation as of where within the genome the genomic selection experiment would induce differentiation in DNA methylation and, as such, did not want to limit our analysis to a reduced and bias representation of the genome.

We used blood samples, rather than tissues of obvious relevance for reproduction (e.g., tissues within the HPGL axis). While information on DNA methylation in blood per se is of little relevance for reproductive functioning, DNA methylation in blood can reflect DNA methylation in other tissues, when DNA methylation is induced in a tissue‐general manner (Lindner, Verhagen, et al., 2021). In red blood cells and liver samples of great tits, (between‐individual) change in DNA methylation over time was established in a tissue‐general and tissue‐specific manner, indicating that, at specific CpG sites, DNA methylation can indeed change in a tissue‐general manner (Lindner, Verhagen, et al., 2021). However, DNA methylation is not exclusively established in a tissue‐general manner which means that these findings cannot be generalized and that more between‐tissue comparisons are needed to establish in which context(s) DNA methylation patterns in blood can reflect DNA methylation patterns in other tissues. Furthermore, the functional relevance for DNA methylation on altering gene expression is time‐ and context‐dependent (Laine et al., 2016; Stevenson & Prendergast, 2013). In the great tit, low levels of CpG site methylation (~20%) close to the transcription start site of genes are sufficient to shut down gene expression, while moderate levels of CpG site methylation (up to 60%) within the gene body allow for high gene expression (up to 1000 FPKM; Laine et al., 2016). Furthermore, DNA methylation can be variable over time (Lindner, Laine, et al., 2021; Stevenson & Prendergast, 2013; Viitaniemi et al., 2019) and as such could lead to temporal variation in the expression of affected genes. However, specifically in the case of temporarily variable DNA methylation, DNA methylation may not exclusively act as a cause of gene expression, but can be a result of downstream consequences of gene expression or phenotypes. For example, *Mycobacterium tuberculosis* infection of human dendritic cells is accompanied by changes in CpG site methylation overlapping distal enhancer elements, but changes in CpG site methylation are preceded by changes in gene expression, indicating that identified changes in CpG site methylation might be a downstream consequence of transcriptional activation (Pacis et al., 2019). Due to the complexity underlying the effects of DNA methylation on gene expression (and likely vice versa), we do not know whether the identified DMS indeed affect the expression of genes and in which tissues they may have an effect. On a similar note, identified outlier SNPs can have cis‐ and/or trans‐effects on gene expression and above we discussed the identified SNP in the context of cis‐effects. Assessing the functional relevance of DMS and outlier SNPs on gene expression would require samples and gene expression profiling of the relevant tissues (e.g., tissues within the HPGL axis) which was outside the scope of this study. Furthermore, establishing causal links between DMS or outlier SNPs and lay dates would require experimental validation using functional tools, but, at this point, the application of such functional tools is not feasible in vertebrate non‐model organisms such as the great tit.

## 5 CONCLUSION

We observed genetic and epigenetic differentiation between females from genomic selection lines for early and late avian lay date. Biological functions of genes with such differentiation hint towards a complementary function of DNA methylation and genetic variants, such that DNA methylation might act downstream of the neuro‐endocrine system underlying lay dates, while genetic variants might act at the activation of the neuro‐endocrine system. However, functional validation is required to establish whether identified genetic and epigenetic differentiation is affecting gene expression and reproductive functioning. Nevertheless, the genomic selection experiment for avian lay dates provides insights into where within the genome heritable genetic variation for lay date resides and shows that a part of this variation might be reflected by epigenetic variants.

## CONFLICT OF INTEREST STATEMENT

The authors have no conflict of interest to declare.

## Supporting information


Data S1.

## Data Availability

WGBS data are available at the NCBI BioProject database (http://www.ncbi.nlm.nih.gov/bioproject/) under BioProject PRJNA208335 and accession numbers SRR15410225 to SRR15410232 and SRR21783914 to SRR21783973. Scripts for the bioinformatic pipelines and statistical analyses are published on GitHub (https://github.com/MLindner0/GenomicSelection_AvianLayDate_WGBS).
