# Supplementary material for: Genetic and epigenetic differentiation in response to genomic selection for avian lay date
Source: Evol Appl. 2024 Jun 28;17(7):e13703. doi: 10.1111/eva.13703 (PMC11211926; doi:10.1111/eva.13703)
Supplement: Supplementary file 1 — Data S1. [file EVA-17-e13703-s001.zip › eva13703-sup-0001-Supinfo.docx]

Supplementary material

Genetic and epigenetic differentiation in response to genomic selection for avian lay date

Supplementary Figures


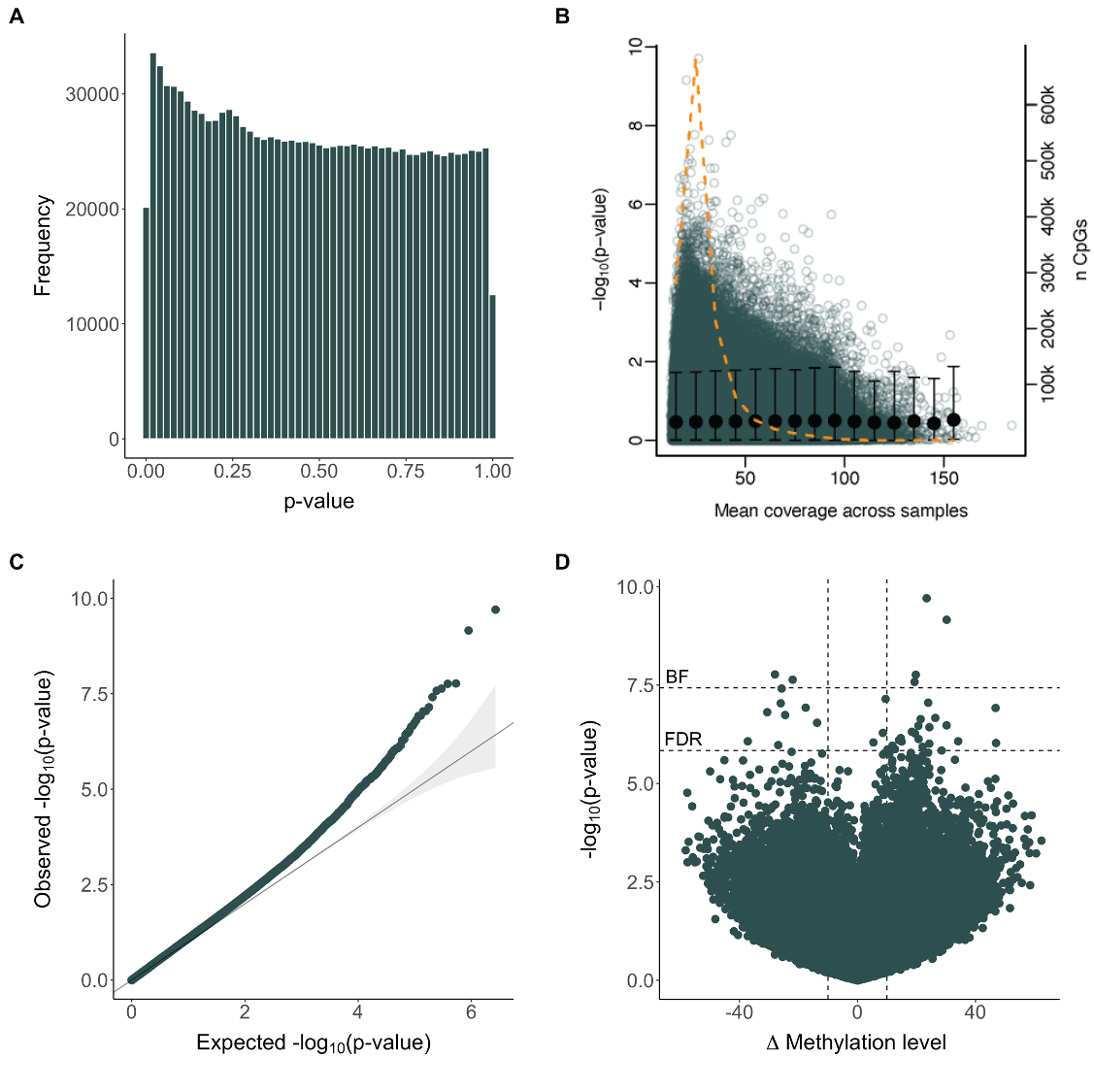


**Figure S1**. Validation of differential methylation analysis with individual samples when testing for a difference between the early and late selection line for lay date. P-value distribution (**A**). Significance vs. mean coverage across samples per CpG site including the mean significance (mean p-value (in log_10_ scale) with 95% confidence intervals (black spheres and error bars) and the number of samples (yellow dashed line) within non-overlapping mean coverage windows of size 10 (i.e. the first window includes CpG sites with mean coverage of 10 to 20 and the last window includes CpG sites with mean coverage of 150 to 160) (**B**). Quantile-quantile-plot (QQ-plot) showing observed p-values (in log_10_ scale) vs. expected p-values (in log_10_ scale) with lambda of 1.15 (**C**). Significance vs. difference in methylation level (calculated by subtracting the mean methylation level of early individuals from the mean methylation level of late individuals for each CpG site (**D**). Vertical dashed lines correspond to a mean difference in methylation level of -10% (left) and 10% (right) and horizontal dashed lines correspond to the significant threshold using Bonferroni (BF, top) and false discovery rate (FDR, bottom) correction for multiple testing.

**Figure S2.** Multi-dimensional scaling (MDS) of SNP data (F3 generation selection line females). Samples are differentiated by colour for females from the early (dark orange) and late (grey-blue) selection line.

**Figure S3**. Trace and density plots of the parameters estimated with BayeScan which include the loglikelihood and the local population F_st_ coefficients.


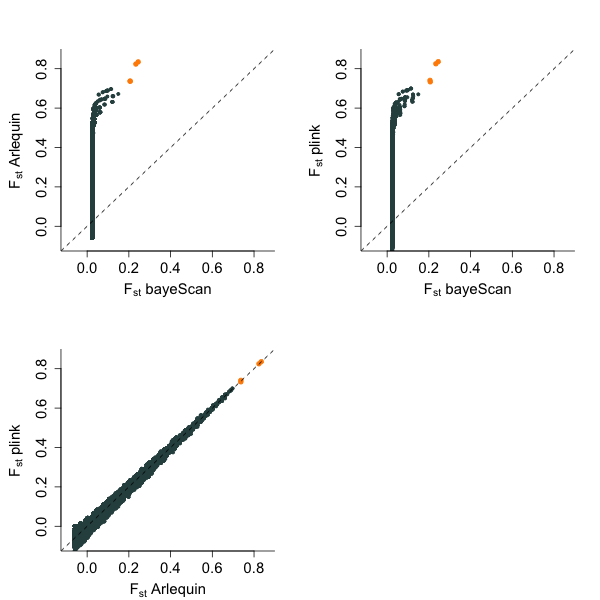


**Figure S4**. Pairwise comparison of F_st_ coefficients estimated with BayeScan, Arlequin and plink. Locus-specific F_st_ coefficients correspond to potential genetic differentiation in response to genomic selection for early and late lay dates. Symbols highlighted in yellow and with increased symbol size are SNPs identified as F_st_ outliers with BayeScan (q-value<0.05). Dashed line corresponds to a correlation of 1.

**Figure S5.** **Distribution of CpG sites over genomic locations.** Genomic regions in which the 1,334,373 CpG sites from the processed methylation data were located. Please note, that genomic regions of the same and different genes might overlap which is why some CpG sites are included more than once (i.e., the sum of CpG sites in the plot is larger than the number of CpG sites in the data set). ‘k’ in y-axis tick labels represents a factor of 1,000. For a definition of the genomic regions see *Annotation of CpG sites and SNPs* in the *Methods* section.
